# *Vernonia britteniana* Root Phytochemical Studies, In Vitro Cercaricidal Activity on the Larval Stage of *Schistosoma mansoni* and Antioxidant Activities

**DOI:** 10.3390/plants12091788

**Published:** 2023-04-27

**Authors:** Maria dos Anjos Valente, Pedro Ferreira, Katelene Lima, Isabel B. Moreira da Silva, Paula Nobre, Isabel Neto, Mavilde Pires, Berta São Braz, Rita Serrano, Silvana Belo, Olga Silva

**Affiliations:** 1 Research Institute for Medicines (iMed.ULisboa), Faculty of Pharmacy, Universidade de Lisboa, Av. Professor Gama Pinto, 1649-003 Lisbon, Portugal; 2 Instituto de Investigação Veterinária, Bairro Santo António, Huambo 555, Angola; 3 Global Health & Tropical Medicine, Medical Parasitology Unit, Universidade Nova de Lisboa, R. da Junqueira 100, 1349-008 Lisbon, Portugal; 4 C.I.I.S.A.—Centre for Interdisciplinary Research in Animal Health, Faculty of Veterinary Medicine, University of Lisbon, Av. Universidade Técnica, 1300-477 Lisbon, Portugal; 5 Associate Laboratory for Animal and Veterinary Sciences (AL4AnimalS), 1300-477 Lisbon, Portugal

**Keywords:** Angola, antioxidant activity, cercaricidal activity, medicinal plant, *Schistosoma mansoni*, traditional medicine, *Vernonia britteniana*, vernoamyoside D

## Abstract

*Vernonia britteniana* Hiern. (*Asteraceae*) is a medicinal plant used in traditional Angolan medicine against schistosomiasis. Our study aimed to investigate the phytochemical composition and the cercaricidal and antioxidant activities in vitro of a traditional herbal preparation (Water-Vbr) and a 70% hydroethanolic extract (EtOH70%-Vbr) prepared with this medicinal plant. The activity of the extracts against *Schistosoma mansoni* cercariae was assessed at different extract concentrations (500, 438, and 125 µg/mL) and at different time intervals, and the phytochemical profiles were obtained by LC-UV-ESI/MS-MS. In addition, the major chemical classes of the identified metabolites were quantified by colorimetry, and the antioxidant potential was assessed using the DPPH and FRAP methods. After 30 min, 100% cercarial mortality was observed at a concentration of 500 μg/mL after exposure, and after 120 min, an LC_50_ of 438 μg/mL was observed for both extracts. Phenolic acid derivatives (chlorogenic acid, caffeic acid; 3,4-di-*O*-caffeoylquinic acid; 3,5-di-*O*-caffeoylquinic acid; and 4,5-di-*O*-caffeoylquinic acid) and triterpenoids (stigmastane-type steroidal saponins; vernoamyoside D and vernonioside D1; vernoamyoside B; and vernoniamyoside A and C) were identified as the main secondary metabolites. The Water-Vbr extract showed the highest antioxidant activity—DPPH: IC_50_ = 1.769 ± 0.049 µg/mL; FRAP: mean = 320.80 ± 5.1325 µgAAE/g.

## 1. Introduction

Over 80% of the African population uses traditional medicine as a primary source of health [1], mainly based on medicinal plants and traditional herbal preparations [2,3,4]. However, according to the latest official Angolan public data, this percentage varies between 30% and 40% in Angola [5]. In this context, medicinal plants are used to treat and prevent various diseases, including parasitic infections such as schistosomiasis [6].

Also known as snail fever, this disease is caused by blood flukes’ parasites of the genus *Schistosoma*, which are responsible for up to 200,000 deaths a year worldwide, with an estimated 230 million infected in 78 countries in Africa, South America, and Southeast Asia [7,8,9]. In addition, it causes losses of up to 4.5 million disability-adjusted life years annually, mainly in sub-Saharan Africa, where over 90% of morbidity and death are concentrated. Among the six schistosome species that infect humans, *Schistosoma haematobium*, the causative agent of urogenital schistosomiasis, is the dominant species on the African continent. In contrast, *Schistosoma mansoni*, one of the five schistosome species responsible for the intestinal form of the disease, is commonly distributed in Africa and South America endemic countries. For example, both types coexist in Angola, resulting in a high disease burden in many rural and suburban areas, with an estimated 5–10% of the population requiring prophylactic chemotherapy [10].

Schistosomiasis transmission to humans occurs when the larval stage of infectious parasites (cercariae) released by freshwater snails penetrates the skin or mucosa after contact with infected water [11]. Development of adult schistosomal worms occurs in the bladder or mesenteric veins, where eggs released from female worms and trapped in tissues induce a wide range of species-specific clinical manifestations in acute and chronic infections [12]. Praziquantel (PZQ) is a synthetic drug used to treat this disease and is the only existing drug for mass administration (MDA) in schistosomiasis control programs. However, because this drug is ineffective against juvenile worms (schistosomula), several schistosomiasis infections go untreated. In addition, the widespread use of PZQ in MDA has raised concerns about the emergence of PZQ-resistant schistosomes, as demonstrated by field isolates from regions involved in extensive MDA programs. Therefore, new therapeutic options are needed to treat this neglected disease [13,14].

Reports on the biological activity of various medicinal plants from the *Vernonia* Schreb. genus (*Asteraceae*), describe their anthelminthic, anti-inflammatory, antioxidant, antimicrobial, cytotoxic [15,16,17], antitumor and anticancer [18], renoprotective [19], antimalarial [20], antituberculous [21], antiparasitic [22] and antifungal activities [23]. In addition, various secondary metabolites, such as phenolic acid derivatives, including flavonoids, hydrolyzable and condensed tannins, lactone sesquiterpenes, and steroidal saponins, have been identified in this genus [24,25,26,27]. 

*Vernonia britteniana* Hiern. is an endemic species to Angola, and local people traditionally use dried root crops to treat schistosomiasis. 

The results of preliminary work on two root extracts of *Vernonia britteniana* indicate interest in investigating the benefits of this medicinal plant for the treatment of schistosomiasis [28]. The present work aims to evaluate the cercaricidal activity in vitro of a traditional herbal preparation and a 70% hydroethanol extract of this herbal medicine.

For each *V. britteniana* extract tested, quantification of the major chemical classes of secondary metabolites was performed, and chemical fingerprinting was determined by high-performance liquid chromatography (LC) coupled with an ultraviolet photodiode array (UV) detector and electrospray tandem mass spectrometry (ESI-MS/MS). In addition, the antioxidant activity of these two herbal preparations was determined.

## 2. Results

### 2.1. Chemical Studies

#### 2.1.1. Drug-Extract Ratio

The drug-extract ratios were 1:5 for the traditional herbal preparations (Water-Vbr) and 1:7 for the 70% hydroethanolic extract (EtOH70%-Vbr).

#### 2.1.2. LC-UV-ESI/MS-MS Chemical Profile

**Figure 1:**
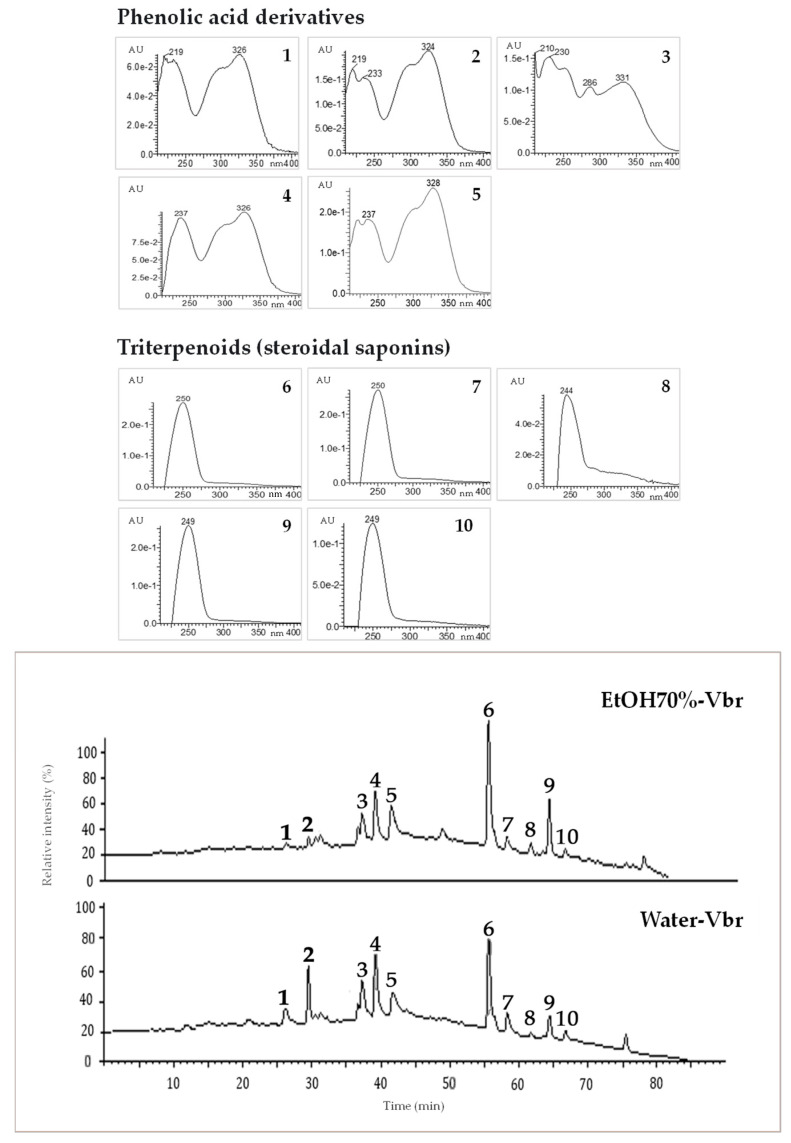
*V*. *britteniana* root extracts’ LC/UV *maxplot* chromatograms (*λ* = 220–410 nm) and UV spectral data of main marker secondary metabolites.

The results of *V. britteniana* root Water-Vbr and EtOH70%-Vbr extract analyzed by LC-UV-ESI/MS-MS are shown in Figure 1 and Table 1. Based on the chromatographic and spectral data, a total of 10 compounds were identified in both extracts, namely one phenolic acid (peak 2), four caffeoylquinic acid derivatives (peaks 1 and peaks 3 to 5), and five triterpenoids (steroidal saponins) (peaks 6 to 10). Obtained data analysis, together with co-chromatography with authentic standards of the commercially available compounds (peaks 1 to 5) and literature data [29,30,31,32,33,34,35], enabled the unequivocal identification of chlorogenic acid (peak 1); caffeic acid (peak 2); 3,4-di-*O*-caffeoylquinic acid (peak 3); 3,5-di-*O*-caffeoylquinic acid (peak 4); and 4,5-di-*O*-caffeoylquinic acid (peak 5) as marker compounds on both extracts. Compared to the reported literature data, peak 6 was tentatively identified as vernoamyoside D [36,37], peak 7 as vernonioside D1 [36], peak 8 as vernoamyoside B [36], peak 9 as vernoniamyoside A [36,37,38], and peak 10 as vernoniamyoside C [36,37,38]. Vernoamyoside D was identified as the principal constituent on both *V. britteniana* root Water-Vbr and EtOH70%-Vbr extracts.

U—absorbance units; EtOH70%-Vbr—*V*. *britteniana* root 70% hydroethanolic extract; nm—nanometers; *V*. *britteniana* root Water-Vbr—aqueous extract.

#### 2.1.3. Secondary Metabolites Quantification

The total content of phenols, triterpenoids, and saponins of *V. britteniana* root Water-Vbr and EtOH-70%-Vbr extracts is presented in Table 2. Both extracts contained saponins as the major classes of secondary metabolites. The Water-Vbr extract showed higher concentrations of all three classes of components relative to the EtOH-70%-Vbr extract. Using the two-tailed unpaired *t*-test, no statistically significant differences (*p* > 0.05) were found between these extracts’ total phenols and triterpenoids content. However, statistically significant differences (*p* = 0.0178) were found between the total saponins content of these extracts. Since saponins are classified as triterpenoids biosynthetically formed from six isoprene units that share the acyclic C_30_ precursor squalene, and no other type of triterpenoid has been identified in this medicinal plant to date, we conclude that the total triterpenoid content is mainly due to this kind of secondary metabolites [39].

### 2.2. Biological Activity

#### 2.2.1. Antioxidant Activity

The results of the evaluation of the antioxidant activity of the Water-Vbr and EtOH70%-Vbr extracts from *V. britteniana* root are presented in Table 3. Both tested extracts showed antioxidant activity, and no statistically significant activity differences were found between these extracts (Water-Vbr *p*-value > 0.05 vs. EtOH70%-Vbr in both tests). However, in the DPPH assay, the anti-radical activity of both extracts was more than 20-fold higher than that of ascorbic acid. Regarding the results of the FRAP assay, both extracts showed similar ferric iron-reducing capacities, with values approaching those of ascorbic acid.

#### 2.2.2. In Vitro Cercaricidal Activity

The lethal concentration of each *V. britteniana* root extract required to eliminate 50% of cercariae (LC_50_) is expressed as the mean and standard deviation. Both extracts, Water-Vbr and EtOH70%-Vbr, showed cercaricidal activity against the larval stage of *S. mansoni*; the cercariae lacked motility and were deposited at the bottom of the cavity, indicating that they were dead, as shown in Table 4. After 120 min of contact, an LC_50_ value of 438 µg/mL was obtained for both extracts. 

Table 5 shows the results of visible structural changes in cercaria after treatment with root extracts of *V. britteniana* (Water-Vbr and EtOH70%-Vbr). Both extracts induced structural changes after 1 h of incubation at a 500 µg/mL concentration.

Application of the Student’s *t*-test showed no significant differences (*p* = 0.9316) between the mean dead cercariae per time interval by administration of the Water-Vbr and EtOH70%-Vbr extracts. Referring to the mean cercariae killed at a concentration of 438 µg/mL, the unpaired *t*-test for independent samples was *t*(df 4) = 0.4472, with a *p*-value = 0.678.

## 3. Discussion

Despite efforts to reduce the burden of disease caused by schistosomiasis, it is still the second most important parasitic disease after malaria with significant public health implications in endemic areas of the tropics and subtropics. It is estimated that approximately 785 million people lack access to essential water services, with over 2 billion lacking access to basic sanitation and 3 billion lacking clean water supplies at home [40]. As of 2019, approximately 36% of Angola’s population lived below the poverty line and faced difficulties in accessing basic public services [40]. The World Health Organization Strategic Cooperation Plan (2015–2019) reported that loiasis, lymphocytic filariasis, and schistosomiasis are still of concern in Angola, with an estimated 12 million people at risk of infection and 2.5 million people requiring treatment [41]. As recommended by this entity, praziquantel is the drug also used in Angola for treatment and in MDA control programs. However, due to its use in monotherapies, as well as its ineffectiveness against juvenile worms and the increasing reports of PZQ resistance or loss of sensitivity of parasite isolates [42], there is an urgent need for new alternative treatments [13,43]. Therefore, the use of medicinal plants with pharmacological properties has been considered to be an alternative to treat various diseases such as cancer [18], malaria [20], tuberculosis [21], trypanosomiasis [44], and even schistosomiasis [45].

Several *Vernonia* species from the African flora are used as an integral part of traditional African medicine. A literature review study conducted by Toyang and Verpoorte (2013) reported that 109 species of *Vernonia* were used as herbal medicines [46]. Results from in vivo and in vitro biological studies help validate their specific medical utility. Among them, *Vernonia amygdalina* Del., an African species traditionally used against schistosomiasis, has long been studied for its chemical composition and biological activity, including anti-schistosomiasis activity. Sesquiterpene lactones and steroidal glucosides isolated from this species have been hypothesized to have anti-schistosomal activity [38]. Acheampong et al. (2020) demonstrated the cercaricidal and adulticidal activity of a *V. amygdalina* whole plant methanol extract against *S. mansoni* as a function of time and concentration. This extract (3 h IC_50_ = 35.84 µg/mL) exerted a high cercaricidal activity. The in vivo recovery of the worms after treatment with the plant extract and praziquantel was 48.8% and 59.9%, respectively (*p* < 0.05), and the mice treated with this extract had lower mean liver and spleen weights compared to those in the untreated groups (*p* < 0.05) and were considered by the authors to be strongly adulticidal in vivo [45]. In addition, aqueous and ethanolic extracts of this medicinal plant with an LC_50_ value of 338.8 ppm and an LC_90_ value of 614.8 ppm proved to be toxic for adult *Biomphalaria pfeifferi* [45]. The molluscicide whole aqueous and ethanolic extracts of *V. amygdalina* are also shown against *Bulinus globosus* with LC_50_ values of 534 ppm and 208 ppm, respectively [47]. 

To our knowledge, no studies on the cercaricidal effect of *V. britteniana* root herbal preparations in the larval stage of *S. mansoni* have been carried out by others. According to our results, the detected activity (LC50 value of 438 g/mL) seems to be lower than the cercaricidal activity of the *V. amygdalina* whole plant methanol extract cited above [45]. However, *V. britteniana* root is explicitly used in traditional Angolan medicine to treat schistosomiasis. Furthermore, no studies on the cercaricidal effect of roots of other *Vernonia* species were found in the literature.

Consistent with chemotaxonomic data from its botanical genus, we identified phenolic acid derivatives (chlorogenic acid; caffeic acid; 3,4-di-*O*-caffeoylquinic acid; 3,5-di-*O*-caffeoylquinic acid; and 4,5-di-*O*-caffeoylquinic acid) and steroid saponins of the stigmastane type (vernoamyoside D and vernonioside D1; vernoamyoside B; and vernoniamyoside A and C) in *V. britteniana* root. 

Phenolic acids, flavonoids, lactone sesquiterpenes, and steroid saponins) are examples of secondary metabolites identified in *V. amygdalina* that have anthelmintic properties [48]. The steroid saponins identified in this species, namely vernonioside A1, A2, A3, and B1 showed anti-schistosomal activity [49]. The major marker compounds, vernoamyoside D, vernonioside D1, vernoamyoside B, vernoniamyoside A, and vernonimyoside C, now identified on *V. britteniana* root might also be involved in the cercaricidal activity of both water and EtOH70% tested extracts.

It has also been reported that dicaffeoylquinic acids are involved in the anti-schistosomal activity of various medicinal plants such as the leaf extracts and branches of *Artemisia annua* L. and *Artemisia afra* Jacq. ex Willd [50]. These classes of secondary metabolites have also been recognized as antioxidants and anti-radical agents [30,34]. 

The antioxidant activity of chlorogenic acid; caffeic acid; 3,4-di-*O*-caffeoylquinic acid; 3,5-di-*O*-caffeoylquinic acid; and 4,5-di-*O*-caffeoylquinic acid isolated from *Vernonia anthelmintica* seed and from *V. amygdalina* leaf has been demonstrated [30,51]. 

The antioxidant activity of the ethanolic extract of *Vernonia patula* Merril’s whole plant was demonstrated using the DPPH method (IC_50_ value of 36.59 g/mL) [52]. The aqueous extract of *V. amygdalina* leaf also showed antioxidant activity, which gave an IC_50_ value of 1831 ± 0.15 mg/mL by the DDPH method and a mean value of 1.49 ± 0.18 mmol by the FRAP method. The authors justified this activity with the presence of phenolic compounds, such as flavonoids, anthocyanins, and proanthocyanins [53,54,55]. 

Our results showed that both *V. britteniana* extracts have a strong antioxidant potential, which can be attributed to the presence of the identified phenolic acid derivatives and steroidal saponins.

## 4. Materials and Methods

### 4.1. Chemicals and Reagents

Folin–Ciocalteu reagent; catechin; gallic acid; oleanolic acid; vanillin; 2,2-diphenyl-1-picrylhydrazl (DPPH); chlorogenic acid; caffeic acid; 3,4-di-*O*-caffeoylquinic acid; 3,5-di-*O*-caffeoylquinic acid; and 4,5-di-*O*-caffeoylquinic acid were purchased from Sigma-Aldrich (Bangalore, India). Methanol was purchased from Honeywell (Hamburg, Germany). Sodium acetate trihydrate and 70% perchloric acid were purchased from V.W.R. (Leuven, Belgium). Ferric chloride, sodium carbonate, and acetic acid were purchased from Merck (Darmstadt, Germany). Dimethyl sulfoxide (DMSO) was obtained from Carlo Erba (Val-de-Reuil, France). Formic acid and high-performance liquid chromatography (HPLC) solvents (acetonitrile and methanol) were obtained from Fisher Scientific (Merelbeke, Belgium).

### 4.2. Collection and Preparation of Plant Material

**Figure 2:**
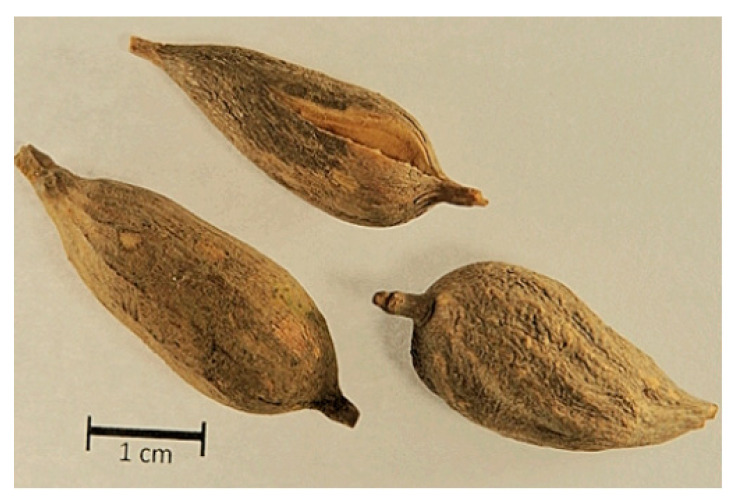
Samples of tuberous roots from *V*. *britteniana*.

The roots of *V. britteniana* (Figure 2) were collected in August 2018 in the Huambo region of Angola in the village of Ndango de Cima (latitude 12°49′17″ S and longitude 15°38′20″ E). After collection, the plant material was dried in the dark and stored in an airy room until further use. Relevant plant parts required for sample identification were also collected, and specimen #LISC13028 was prepared and identified by Maria Cristina Duarte, Scientific Curator of the LISC Herbarium, and from plant collections at the Tropical Botanical Garden and the Lisbon Botanical Garden.

### 4.3. Extract Preparation

#### 4.3.1. Aqueous Extract

The aqueous extract (Water-Vbr) was prepared to reproduce the recipe provided by traditional medicine practitioners in the Huambo region. Twenty-nine grams of dried V. britteniana root were ground into powder using a grinder (Ika, Multidrive, Staufen, Germany) and extracted by maceration with 1 L of water at room temperature for 90 min. After filtration, the obtained solution was frozen and dried in a lyophilized Heto LyoLab-3000 (Dietikon, Switzerland).

#### 4.3.2. 70% Hydroethanolic Extract

The 70% hydroethanolic extract (EtOH70%-Vbr) was prepared by macerating dried root powder with EtOH70% (ratio 1:10, *w/v*) at room temperature for 24 h with stirring. After filtration, the obtained extract was concentrated to dryness under reduced pressure (T 40 °C) using a Buchi Rotavapor R-100 (Flawil, Switzerland) [56].

### 4.4. Phytochemical Studies

#### 4.4.1. LC-UV-ESI/MS-MS Chemical Profile

A Waters Alliance 2695 high-performance liquid chromatography (LC) instrument with autosampler and photodiode array detector (Waters PDA 2996) coupled to a MicroMass Quattromicro API triple quadrupole tandem mass spectrometer (Waters, Drinagh, Ireland). The separation module (Waters, Drinagh, Ireland) comprises a quaternary pump system, degasser, autosampler, and column oven. The chromatograms were recorded at each maximum peak absorbance between the wavelengths of 220 to 410 nm (Maxplot).

The separation was performed on a LiCrospher^®^ 100 RP-18 column 5 µm (250 × 4 mm, Merck); column temperature 35 °C; flow rate 0.3 mL/min; injection volume (20 μL); mobile phase (A) water + 0.1% formic acid/(B) acetonitrile—total time of 90 min under the gradient conditions given in Table 6.

The ionization of the compounds was performed by an electrospray source in positive (ESI^+^) and negative (ESI^−^) modes at different cone voltages (20 to 40 V). MassLynx software version 4.1 was used for data acquisition and processing. 

#### 4.4.2. Total Phenol Content

The phenol content of the Water-Vbr and EtOH70%-Vbr extracts was determined using the Folin–Ciocalteu reagent method [57] with modifications. Briefly, 2 mL of Folin–Ciocalteu reagent (previously diluted 1:10 (*v/v*) in water) was mixed with 1.6 mL of sodium carbonate (75 g/L) after adding 0.4 mL of sample. After 2 h of incubation at room temperature, protected from light, the absorbance at 765 nm was measured using a Hitachi U-2000 spectrophotometer (Tokyo, Japan). Gallic acid was used as a standard control. Three replicates were performed to obtain the measurements, and the mean and standard deviation values were fitted using the straight-line equation (Y = 00.0016x + 0.0644 and R = 0.9936). Results are presented as mean (±SD) gallic acid equivalents per gram of extract.

#### 4.4.3. Total Triterpenoid Content

The total triterpenoid content of the Water-Vbr and EtOH70%-Vbr extracts was determined using a colorimetric method [58] with modifications. Briefly, 100 μL of the extract was mixed with vanillin/glacial acetic acid (150 μL, 5% (*p/v*)) and perchloric acid solution (500 μL). The sample solutions were heated at 60 °C for 45 min and then cooled to room temperature. Subsequently, 2.25 mL of glacial acetic acid was added, and the absorbance was measured at 765 nm using a spectrophotometer Hitachi U-2000 (Tokyo, Japan). Oleanolic acid was used as the standard control. Measurements were obtained in triplicate, and the mean and standard deviation values were adjusted using a straight-line equation (Y = 0.0013x + 0.0858 and R^2^ = 0.9991). The results are presented as the mean (±SD) of oleanolic equivalents per gram of the extract.

#### 4.4.4. Saponins Index (SI) 

The saponins content of the Water-Vbr and EtOH70%-Vbr extracts was determined according to the Portuguese Pharmacopeia [59]. Briefly, 1 g of extract was added to 25 mL of alcoholic potassium hydroxide solution (0.5 M) with some glass beads and then heated for 30 min. Then 1 mL of phenolphthalein solution was added, followed by titration with 0.5 M hydrochloric acid (1 mL). A blind test was also carried out under the same conditions. The quantification was based on the formula: SI = 28.05 (n1 − n2)/m.

### 4.5. DPPH Radical Scavenging Activity 

The radical scavenging activity of the Water-Vbr and EtOH70%-Vbr extracts was evaluated using the 2,2-diphenyl-1-picrylhydrazl (DPPH) method [60] with modifications. First, the DPPH solution (3.9 mL, 6 × 10^−5^ M) in methanol was mixed with 100 μL of the diluted extract. After incubation at room temperature for 30 min, the absorbance at 517 nm was measured using a Hitachi U-2000 spectrophotometer (Tokyo, Japan). Ascorbic acid was used as a standard control. The scavenging activity of DPPH was calculated using the following formula: % scavenger = (control absorbance, test sample absorbance/control absorbance) × 100. Results are expressed as mean (±SD) and presented as IC_50_ values.

### 4.6. Ferric-Reducing Antioxidant Power (FRAP) Activity

The iron (III)-reducing ability of the Water-Vbr and EtOH70%-Vbr extracts was assessed using the Ferric-Reducing Antioxidant Power Assay (FRAP) [61] with modifications. Briefly, 100 μL of the extract was mixed with 3 mL of FRAP reagent (25 mL acetate buffer, 2.5 mL of 2,4,6-tripyridyl-s-triazine, and 2.5 mL FeCl_3_). 6H2O solution in the portion (1:1:10). After incubation at 37 °C for 4 min, the absorbance at 593 nm was measured using a Hitachi U-2000 spectrophotometer (Tokyo, Japan). Ascorbic acid was used as a standard. Three replicates were performed to obtain the measurements, and the mean and standard deviation values were fitted using a linear equation (Y = 0.001x + 0.0132 and R = 0.9944). Results are presented as mean (±SD) ascorbic acid equivalents per gram of extract.

### 4.7. Statistical Analysis

Data analysis for total phenol and triterpene content was performed using Microsoft Excel. Results were expressed as the mean (±SD) with units expressed in equivalents of the standards used for each assay. The two-tailed unpaired Student *t*-test was used to verify the detection of significant differences between the mean content of phenols, triterpenoids, and saponins and the antioxidant activity of Water-Vbr and EtOH70%-Vbr extracts. A *p* < 0.05 was considered significant Water-Vbr vs. EtOH 70%-Vbr extracts.

### 4.8. In Vitro Cercaricidal Activity of V. britteniana Root

#### 4.8.1. Sample Preparation

Extemporaneous solutions of each extract (concentration 20 mg/mL) were prepared and used to determine the cercaricidal effect on *S. mansoni* larvae. First, the aqueous extract was diluted in deionized water, and the hydroethanolic extract was diluted in 1% DMSO. Then the samples were agitated in an ultrasonic bath (BRANSON 3200, USA).

#### 4.8.2. Obtaining Live *S. mansoni* Cercariae

The assay was performed on live cercariae obtained from the freshwater snail *Biomphalaria glabrata* (Belo Horizonte, Brazil strain) previously infected with a strain of *S. mansoni* (Belo Horizonte, Brazil strain), routinely maintained in Mus musculus CD1 male mice at IHMT-UNL.

About eight freshwater snails were placed in a glass beaker containing 10 mL of distilled water and exposed to an artificial light source (70 W lamp) to monitor the release of cercariae by visual observation under a stereomicroscope (4×). All cercaria were placed in a single container. After homogenization, 500 µL of the solution was dispensed onto five glass slides for counting.

#### 4.8.3. Determination of Cercaricidal Activity

The surrounding activity was determined according to the protocol described by Tekwu and co-workers (2017) [62]. In 24-well plates (Polystyrene, Gdynia, Spain), 1 mL of a solution containing approximately 50 cercariae was added to each well. The samples were tested at 125, 438, and 500 g/mL concentrations in 1.5 mL total solution volume. After homogenization with a round stirrer (New Brunswick Scientific, Classic model, Edison Township, NJ, USA), cercariae survival and mortality were observed at intervals of 30 to 120 min incubation using an inverted optical microscope (Olympus CKX41, Japan 4×) and magnification of 10×, 20× and 40×. A positive control (praziquantel 10 µg/mL solution; Thermo Fisher Kandel, Lenzkirch, Germany) and a negative control (distilled water + 1% DMSO) were used in each assay. Mobility, mortality, and structural damage to the cercariae were assessed. Cercariae were considered dead if deposited on the bottom of the well without movement during the time intervals evaluated (30, 60, 90, 120, and 150 min). Altering and destroying the cercaria, such as by severing and crushing their tail, has been considered structural damage. LC_50_ was determined using the geometric mean of the cercariae count after exposure of each sample to the different concentrations for 2 h and 30 min. All experiments were performed in triplicate.

#### 4.8.4. Statistical Analysis

The data obtained were analyzed using Microsoft Excel software, and the results were expressed as the mean (±SD). An unpaired, two-tailed Student *t*-test was used to verify the detection of significant differences between the means of the results from the two extracts. A value of *p* < 0.05 was considered significant concerning Water-Vbr vs. EtOH70%-Vbr extracts.

## 5. Conclusions

Schistosomiasis is an endemic parasitic disease that is widespread in Africa, particularly in the sub-Saharan region including Angola [10].

The use of traditional medicinal plant preparations is recognized as an added value in combating and eliminating this disease. For the first time, the usefulness of *V. britteniana* root in schistosomiasis has been evaluated and the major classes of secondary metabolites have been characterized. Phenolic acid derivatives (chlorogenic acid; caffeic acid; 3,4-di-*O*-caffeoylquinic acid; 3,5-di-*O*-caffeoylquinic acid; and 4,5-di-*O*-caffeoylquinic acid) and steroid saponins (vernoniamyoside A and C, vernoamyoside D, vernonioside D1 and vernoamyoside B) were the main components of this medicinal plant. Its possible correlation with the in vitro cercaricidal and antioxidant activities verified with both Water-Vbr and EtOH70%-Vbr extracts will be investigated in the future along with additional studies on the quality, biological activity (against adult worms and eggs), and safety of this medicinal plant. 

The present study underlines the importance of ethnopharmacological studies to validate the usefulness of traditional medicines.

## Figures and Tables

**Table 1:** LC-UV-ESI/MS-MS identification of main marker *V. britteniana* root secondary metabolites.

Peak N°	*t*_r_ (min)	UVλ_max·_ (nm)	[M-H]^−^ or [M + H]^+^ (*m/z*)	MS-MS Fragment Ions (*m*/*z*)	Assignment
1	26.31	326, 332	353 [M-H]^−^	191, 179	chlorogenic acid
2	29.64	324, 233	179 [M-H]^−^	135, 89	caffeic acid
3	36.81	331, 230	515 [M-H]^−^	191, 135	3,4-di-*O*-caffeoylquinic acid
4	39.33	326, 237	515 [M-H]^−^	353, 191, 179	3,5-di-*O*-caffeoylquinic acid
5	41.96	328, 237	515 [M-H]^−^	353, 191, 179	4,5-di-*O*-caffeoylquinic acid
6	56.81	250	649[M + H]^+^	631[M + H-H_2_O]^+^ 487[M + H-H_2_O + Glc]^+^ 469[M + H-Glc]^+^	vernoamyoside D *
7	58.74	250	649 [M + H]^+^	631[M + H-H_2_O]^+^ 469[M + H-Glc]^+^ 451[M + H-H_2_O-Glc]^+^	vernonioside D1 *
8	61.59	244	649 [M + H]^+^	487[M + H+H_2_O-Glc]^+^ 469 [M + H-Glc]^+^	vernoamyoside B *
9	64.66	249	647 [M + H]^+^	485[M + H+H_2_O-Glc]^+^ 467 [M + H-Glc]^+^	vernoniamyoside A *
10	66.80	249	647[M + H]^+^	485[M + H+H_2_O-Glc]^+^ 467[M + H-Glc]^+^	vernoniamyoside C *

* Tentatively assigned based on literature data [34,35,36,37,38]; EtOH70%-Vbr—70% hydroethanolic extract; Glc—glucose; MS-MS—mass spectrometry; *m/z*—mass-to-charge ratio; [M-H]^−^—negative mass electrospray ionization mode; [M-H]^+^—positive mass electrospray ionization mode; *t*_r_—retention time; UVλ_max·_—wavelength of maximum absorbance; Vbr—*V. britteniana* root; Water-Vbr—aqueous extract.

**Table 2:** Quantification of the main class of secondary metabolites of *V. britteniana* root.

Class of Secondary Metabolites	Extract	
	Water-Vbr	EtOH70%-Vbr
Total phenols (mg GAE/g extract)	139.750 ± 3.704 *	102.875 ± 1.347
Total triterpenoids (mg OAE/g extract)	167.077 ± 2.643 *	153.231 ± 16.667
Saponins Index (g extract)	370.3 ± 24.450	296.0 ± 22.670

EtOH70%-Vbr—70% hydroethanolic extract; GAE—gallic acid equivalents; OAE—oleanolic acid equivalents; SD—standard deviation; Vbr—*V*. *britteniana* root; Water-Vbr—aqueous extract; * *p*-value > 0.05 Water-Vbr vs. EtOH70%-Vbr.

**Table 3:** Antioxidant activity of *V. britteniana* root.

Extract	Essay	
	DPPH IC50 ± SD (µg/mL Extract)	FRAP Mean ± SD (µg AAE/g Dried Extract)
Water-Vbr	1.769 ± 0.049	320.800 ± 5.132
EtOH70%-Vbr	2.928 ± 0.138	286.800 ± 4.780
Ascorbic acid-AAE	67.446 ± 0.746	256.800 ± 5.706

AAE—ascorbic acid equivalent; EtOH70%-Vbr—70% hydroethanolic extract; IC_50_—half of the maximum inhibitory concentration; SD—standard deviation; Vbr—*V*. *britteniana* root; Water-Vbr—aqueous extract.

**Table 4:** LC_50_ of *V. britteniana* root on *S. mansoni* cercariae after 120 min in vitro exposure.

Extract	LC50 (µg/mL)	Time (min)	Mean No. of Dead Cercariae (µg/mL)
Water-Vbr	438	120	25.7 ± 1.16
EtOH70%-Vbr	438	120	25.3 ± 0.58
PZQ	10	120	50

EtOH70%-Vbr—70% hydroethanolic extract; LC_50_—lethal concentration that kills 50% of the cercariae; PZQ—Praziquantel, positive control; SD—standard deviation; Vbr—*V*. *britteniana* root; Water-Vbr—aqueous extract.

**Table 5:** Cercariae mortality and structural changes observed after incubation with *V. britteniana* root.

Extract	Concentration (µg/mL)	Average Cercaria Killed					Structural Changes				
		Observation Time (min)					Observation Time (min)				
		30	60	90	120	150	30	60	90	120	150
Water-Vbr	125	0	0	0	0	0	0	-	-	-	-
	438	0	17.7	21.0	25.7	0	-	-	-	-	-
	500	50	0	0	0	0	-	+	-	-	-
EtOH70%-Vbr	125	0	0	0	0	0	-	-	-	-	-
	438	0	15.0	20.7	25.3	0	-	-	-	-	-
	500	50	0	0	0	0	0	+	-	-	-

0—no mortality; (-)—no structural changes observed; (+)—structural changes observed; EtOH70%-Vbr—70% hydroethanolic extract; Vbr—*V*. *britteniana* root; Water-Vbr—aqueous extract.

**Table 6:** *V. V. britteniana* LC-UV-ESI/MS-MS chromatography gradient used.

Time (min)	%A	%B
0.00	95	5
10.00	95	5
30.00	82	18
44.00	64	36
64.00	64	36
90.00	10	90

## Data Availability

Not applicable.

## References

[1] World Health Organization (2000). Regional Committee for Africa. Promoting the Role of Traditional Medicine in Health Systems: A Strategy for the African Region..

[2] Gasparetto J.C., Martins C.A.F., Hayashi S.S., Otuky M.F., Pontarolo R. (2012). Ethnobotanical and scientific aspects of *Malva sylvestris L*.: A millennial herbal medicine. J. Pharm. Pharmacol..

[3] Bernardini S., Tiezzi A., Laghezza Masci V., Ovidi E. (2018). Natural products for human health: A historical overview of the drug discovery approaches. Nat. Prod. Res..

[4] Newman D.J., Cragg G.M. (2016). Natural products as sources of new drugs from 1981 to 2014. J. Nat. Prod..

[5] Ministério da Saúde (2007). Angola—Despesas Públicas no Sector da Saúde 2000–2007.

[6] Simoben C.V., Ntie-Kang F., Akone S.H., Sippl W. (2018). Compounds from African medicinal plants with activities against selected parasitic diseases: Schistosomiasis, trypanosomiasis, and leishmaniasis. Nat. Prod. Bioprospect..

[7] Albuquerque R.D.D.G., Mahomoodally M.F., Lobine D., Suroowan S., Rengasamy K.R.R. (2020). Botanical products in the treatment and control of schistosomiasis: Recent studies and distribution of active plant resources according to affected regions. Biology.

[8] Neves B.J., Andrade C.H., Cravo P.V.L. (2015). Natural products as leads in schistosome drug discovery. Molecules.

[9] World Health Organization (2023). Fact Sheet Schistosomiasis. https://www.who.int/news-room/factsheets/detail/schistosomiasis.

[10] Mendes E.P., Okhai H., Cristóvão R.E., Almeida M.C., Katondi N., Thompson R., Lopes S. (2022). Mapping of schistosomiasis and soil-transmitted helminthiases across 15 provinces of Angola. PLoS. Negl. Trop. Dis..

[11] Gomes S.E.C., Mesquita M.C.S., Rehn V.N.C., Nascimento W.R.C., Loyo R., Barbosa C.S. (2016). Transmissão urbana da esquistossomose: Novo cenário epidemiológico na zona da mata de Pernambuco. Rev. Bras. Epidemiol..

[12] LoVerde P.T., Toledo R., Fried B. (2019). Schistosomasis. Digenetic Trematodes.

[13] Oliveira R.N., Rehder V.L.G., Oliveira A.S.S., Jeraldo V.D.L.S., Linhares A.X., Allegretti S.M. (2014). Anthelmintic activity in vitro and in vivo of *Baccharis trimera* (Less) DC against immature and adult worms of *Schistosoma mansoni*. Exp. Parasitol..

[14] Vale N., Gouveia M.J., Rinaldi G., Brindley P.J., Gärtner F., Correia da Costa J.M. (2017). Praziquantel for schistosomiasis: Single-drug metabolism revisited, mode of action, and resistance. Antimicrob. Agents. Chemother.

[15] Akoto C.O., Acheampong A., Boakye Y.D., Asante B., Ohene S., Amankwah F. (2021). Anthelminthic, anti-inflammatory, antioxidant, and antimicrobial activities and FTIR analyses of *Vernonia camporum* stem-bark. J. Chem..

[16] Habtamu A., Melaku Y. (2018). Antibacterial and antioxidant compounds from the flower extracts of *Vernonia amygdalina*. Adv. Pharmacol. Pharm. Sci..

[17] Unuofin J.O., Oladipo A.O., Msagati T.A.M., Lebelo S.L., Meddows-Taylor S., More G.K. (2020). Novel silver-platinum bimetallic nanoalloy synthesized from *Vernonia mespilifolia* extract: Antioxidant, antimicrobial, and cytotoxic activities. Arab. J. Chem..

[18] Lowe H.I.C., Daley-Beckford D., Toyang N.J., Watson C., Hartley S., Bryant J. (2014). The anticancer activity of *Vernonia divaricata* Sw against leukaemia, breast and prostate cancers *in vitro*. West Indian Med. J..

[19] Dogra N.K., Kumar S. (2015). A review on ethnomedicinal uses and pharmacology of *Vernonia cinerea* Less. Nat. Prod. Res..

[20] Bihonegn T., Giday M., Yimer G., Animut A., Sisay M. (2019). Antimalarial activity of hydromethanolic extract and its solvent fractions of *Vernonia amygdalina* leaves in mice infected with *Plasmodium berghei*. SAGE Open Med..

[21] Kahaliw W., Aseffa A., Abebe M., Teferi M., Engidawork E. (2017). Evaluation of the antimycobacterial activity of crude extracts and solvent fractions of selected Ethiopian medicinal plants. BMC Complement. Altern. Med..

[22] Panda S.K., Luyten W. (2018). Antiparasitic activity in Asteraceae with special attention to ethnobotanical use by the tribes of Odisha, India. Parasite.

[23] Rustamova N., Gao Y., Zhang Y., Yili A. (2020). Biological activity of endophytic fungi from the roots of the medicinal plant *Vernonia anthelmintica*. Microorganisms.

[24] Gahamanyi N., Munyaneza E., Dukuzimana E., Tuyiringire N., Pan C.H., Komba E.V.G. (2021). Ethnobotany, ethnopharmacology, and phytochemistry of medicinal plants used for treating human diarrheal cases in Rwanda: A review. Antibiotics.

[25] Boadu A., Singh S., Karpoormath R., Nlooto M. (2019). Review on ethnomedicinal uses, phytochemical constituents and pharmacological evidence on leaf extract of *Persea americana* and *Vernonia amygdalina* of the African continent—A Review. Indian Drugs.

[26] Alara O.R., Abdurahman N.H., Ukaegbu C.I., Azhari N.H., Kabbashi N.A. (2018). Metabolic profiling of flavonoids, saponins, alkaloids, and terpenoids in the extract from *Vernonia cinerea* leaf using LC-Q-TOF-MS. J. Liq. Chromatog. Relat. Technol..

[27] Dogra N.K., Kumar S., Kumar D. (2020). *Vernonia anthelmintica* (L.) Willd.: An Ethnomedicinal, phytochemical, pharmacological, and toxicological review. J. Ethnopharmacol..

[28] Valente M., Ferreira P., Belo S., da Silva I.M., Nobre P., Lima K., Neto I., Pires M., Serrano R., Silva O. (2022). In vitro cercaricidal activity and phytochemical profile of *Vernonia britteniana* root. Planta Med..

[29] National Institutes of Health (NIH), National Library of Medicine, PubChem. https://pubchem.ncbi.nlm.nih.gov/.

[30] Johnson C.E., Lin L.Z., Harnly J.M., Oladeinde F.O., Kinyua A.M., Michelin R., Bronner Y. (2011). Identification of the phenolic components of *Vernonia amygdalina* and *Russelia equisetiformis*. J. Nat. Prod..

[31] Sun J., Liang F., Bin Y., Li P., Duan C. (2007). Screening non-colored phenolics in red wines using liquid chromatography/ultraviolet and mass spectrometry/mass spectrometry libraries. Molecules.

[32] Alara O.R., Abdurahman N.H., Ukaegbu C.I. (2018). Soxhlet extraction of phenolic compounds from *Vernonia cinerea* leaves and its antioxidant activity. J. Appl. Res. Med. Aromat. Plants.

[33] Willems J.L., Khamis M.M., Saeid W.M., Purves R.W., Katselis G., Low N.H., El-Aneed A. (2016). Analysis of a series of chlorogenic acid isomers using differential ion mobility and tandem mass spectrometry. Anal. Chim. Acta.

[34] Zheng Z., Wang X., Liu P., Li M., Dong H., Qiao X. (2018). Semi-preparative separation of 10 caffeoylquinic acid derivatives using high speed countercurrent chromatogaphy combined with semi-preparative HPLC from the roots of burdock (*Arctium lappa* L.). Molecules.

[35] Wang Y.H., Meng Y., Zhai C., Wang M., Avula B., Yuk J., Khan I.A. (2019). The Chemical Characterization of *Eleutherococcus senticosus* and Ci-wu-jia Tea using UHPLC-UV-QTOF/MS. Int. J. Mol. Sci..

[36] Vasincu A., Luca S.V., Charalambous C., Neophytou C.M., Skalicka-Woźniak K., Miron A. (2022). LC-HRMS/MS Phytochemical profiling of *Vernonia kotschyana* Sch. Bip. Ex Walp.: Potential involvement of highly-oxygenated stigmastane-type saponins in cancer cell viability, apoptosis and intracellular ROS production. S. Afr. J. Bot..

[37] Zhao M.L., Shan S.J., Tao R., Cui L.T., Li Q.R., Luo J., Li Y. (2021). Stigmastane-type steroid saponins from the leaves of *Vernonia amygdalina* Del. Fitoterapia.

[38] Wang J., Song H., Wu X., Zhang S., Gao X., Li F., Chen Q. (2018). Steroidal saponins from *Vernonia amygdalina* Del. and their biological activity. Molecules.

[39] Mukherjee P.K. (2019). Quality Control and Evaluation of Herbal Drugs: Evaluating Natural Products and Traditional Medicine.

[40] World Health Organization (2021). Ending the Neglect to Attain the Sustainable Development Goals: A Global Strategy on Water, Sanitation, and Hygiene to Combat Neglected Tropical Diseases, 2021–2030.

[41] Organização Mundial da Saúde (2016). Escritório Regional para a África. Estratégia de cooperação da OMS 2015-2019: Angola. Organização Mundial de Saúde. Escritório Regional Africano..

[42] Wang W., Wang L., Liang Y.S. (2012). Susceptibility, or resistance of praziquantel in human schistosomiasis: A review. Parasitol. Res..

[43] Bergquist R., Utzinger J., Keiser J. (2017). Controlling schistosomiasis with praziquantel: How much longer without a viable alternative?. Infect. Dis. Poverty.

[44] Kimani N.M., Matasyoh J.C., Kaiser M., Brun R., Schmidt T.J. (2018). Sesquiterpene lactones from *Vernonia cinerascens* Sch. Bip. and their *in vitro* antitrypanosomal activity. Molecules.

[45] Acheampong D.O., Owusu-Adzorah N., Armah F.A., Aninagyei E., Asiamah E.A., Thomford A.K., Anyan W.K. (2020). Ethnopharmacological evaluation of schistosomicidal and cercaricidal activities of some selected medicinal plants from Ghana. Trop. Med. Health.

[46] Toyang N.J., Verpoorte R. (2013). A review of the medicinal potentials of plants of the genus *Vernonia* (Asteraceae). J. Ethnopharmacol..

[47] Oguche O., Olofintoye L.K. (2018). Molluscicidal effect of *Vernonia amygdalina* (Del) and *Momordica charantia* Linn. on *Bulinus* (Phy) *globosus*. Int. J. Multidiscip. Sci. Eng..

[48] Oyeyemi I.T., Akinlabi A.A., Adewumi A., Aleshinloye A.O., Oyeyemi O.T. (2018). *Vernonia amygdalina*: A folkloric herb with anthelminthic properties. Beni. Suef. Univ. J. Basic Appl. Sci..

[49] Jisaka M., Ohigashi H., Takagaki T., Nozaki H., Tada T., Hirota M., Koshimizu K. (1992). Bitter steroid glucosides, vernoniosides A1, A2, and A3, and related B1 from a possible medicinal plant, *Vernonia amygdalina*, used by wild chimpanzees. Tetrahedron.

[50] Taljaard L., Probst A., Tornow R., Keiser J., Haynes R.K., van der Kooy F. (2022). *In vitro* antischistosomal activity of *Artemisia* annua and *Artemisia afra* extracts. Phytomed. Plus.

[51] Bian G.L., Hu Y.L., Yan K., Cheng X.J., Li D.Q. (2022). Characterization of constituents by UPLC-MS and the influence of extraction methods of the seeds of *Vernonia anthelmintica* Willd.: Extraction, characterization, antioxidant, and enzyme modulatory activities. Heliyon.

[52] Lyzu C., Mitra S., Perveen K., Khan Z., Tareq A.M., Bukhari N.A., Dashti M.G. (2022). Phytochemical profiling, antioxidant activity, and in silico analyses of *Sterculia villosa* and *Vernonia patula*. Evid. Based Complement. Altern. Med..

[53] Omede A., Suleiman M.S., Atanu F.O., Momoh S., Friday E.T., Sheneni V.D., Jegede E.R. (2018). Evaluation of antioxidant and cytotoxic properties of *Vernonia amygdalina*. Int. J. Cell Sci. Mol. Biol..

[54] Alara O.R., Abdurahman N.H., Olalere O.A. (2018). Ethanolic extraction of bioactive compounds from *Vernonia amygdalina* leaf using response surface methodology as an optimization tool. J. Food Meas. Charact..

[55] Alara O.R., Abdurahman N.H., Olalere O.A. (2018). Optimization of microwave-assisted extraction of flavonoids and antioxidants from *Vernonia amygdalina* leaf using response surface methodology. Food Bioprod. Process..

[56] European Directorate for the Quality of Medicines EDQM (2019). European Pharmacopeia.

[57] Scalbert A., Monties B., Janin G. (1989). Tannins in wood: Comparison of different estimation methods. J. Agric. Food Chem..

[58] Chang C.L., Lin C.S. (2012). Phytochemical composition, antioxidant activity, and neuroprotective effect of *Terminalia chebula* Retzius extracts. Evid. Based Complement. Altern. Med..

[59] Farmacopeia Portuguesa (2009). Métodos Analíticos-Métodos de Doseamento.

[60] Brand-Williams W., Cuvelier M.E., Berset C. (1995). Use of a free radical method to evaluate antioxidant activity. LWT Food Sci. Technol..

[61] Benzie I.F., Strain J.J. (1999). Ferric reducing/antioxidant power assay: Direct measure of total antioxidant activity of biological fluids and modified version for simultaneous, measurement of total antioxidant power and ascorbic acid concentration. Methods Enzymol..

[62] Tekwu E.M., Bosompem K.M., Anyan W.K., Appiah-Opong R., Owusu K.B.A., Tettey M.D., Kissi F.A., Appiah A.A., Penla Beng V., Nyarko A.K. (2017). In vitro assessment of anthelmintic activities of *Rauwolfia vomitoria* (Apocynaceae) stem bark and roots against parasitic stages of *Schistosoma mansoni* and cytotoxicity study. J. Parasitol. Res..

